# Endodontic Treatment of a Maxillary Second Molar With Five Canals: A Case Report and a Literature Review

**DOI:** 10.7759/cureus.59179

**Published:** 2024-04-27

**Authors:** He Liu, Jing Hao, Ya Shen

**Affiliations:** 1 Oral Biological and Medical Sciences, University of British Columbia, Vancouver, CAN; 2 Conservative and Endodontic Dentistry, Hangzhou Stomatology Hospital, Hangzhou, CHN

**Keywords:** dental operating microscope (dom), cone-beam computed tomography, mesial buccal third canal, endodontic treatment, maxillary second molar, root canal treatment (rct)

## Abstract

A thorough understanding of both common and uncommon root canal anatomies is crucial for the success of root canal treatments, as missing a canal can lead to treatment failure. Although the maxillary second molar typically features three canals, the occurrence of five canals, particularly a mesial buccal third canal (MB3) canal in the mesiobuccal root, is extremely rare. This case report documents such a rare occurrence in a maxillary second molar with five canals. With the assistance of a dental operating microscope, all canals were successfully located, and root canal preparation, irrigation, and filling were accomplished. This case report underscores the significance of in-depth knowledge of root canal anatomy and the invaluable aid of a dental operating microscope in achieving successful root canal treatments.

## Introduction

Apical periodontitis (AP) arises as an inflammatory reaction in periapical tissues, typically triggered by bacterial biofilm within the root canal system [1-3]. The efficacy of root canal treatment is heavily reliant on thoroughly eradicating this biofilm [4-6]. Missed canals, often a result of anatomical variations, have a considerable impact on post-treatment AP, as any residual biofilm can lead to the development or continued presence of AP [7]. Hence, a comprehensive grasp of the anatomical intricacies of the root canal system is vital for the success of root canal treatments.

Maxillary second molars present significant treatment challenges due to their complex root and canal morphologies. A cone-beam computed tomography (CBCT) study of the Chinese population reveals that the majority of maxillary second molars typically have three roots and three canals (66.4%) [8]. Four canals were observed in 22.9% of these molars; this configuration commonly includes two canals in the mesiobuccal root and one canal each in the distobuccal and palatal roots [8]. The presence of five canals is extremely rare, occurring in only 1.4% of cases [8].

The mesiobuccal root of maxillary molars may contain one, two, or occasionally even more canals. The presence of a third canal in the mesiobuccal root, referred to as the mesial buccal third canal (MB3), is exceptionally rare [9]. A detailed review of the internal anatomy of maxillary molars revealed that MB3 canals occur in only 0.5% of 12,200 examined maxillary first molars and 0.3% of 4,090 maxillary second molars, highlighting their scarcity [9].

The aim of this case report is to present a rare instance of a maxillary second molar with five root canals, notably featuring an MB3 canal in the mesiobuccal root. This report underscores the challenges associated with treating a maxillary second molar with such complex anatomy and stresses the vital importance of thorough knowledge of root canal anatomy. Additionally, it highlights the essential role of a dental operating microscope in modern endodontic practice to improve treatment outcomes.

## Case presentation

A 21-year-old male Chinese patient presented with a request to fill a carious cavity in the maxillary right second molar. The tooth had received a composite restoration filling many years ago. The patient had not felt pain since the tooth was filled. The patient discovered that the composite resin restoration was lost, and a carious cavity appeared one week ago. The patient's general health condition was good, classified as ASA I, with no signs or symptoms of any systemic diseases. The patient maintained good oral hygiene and did not have any history of deleterious or parafunctional habits.

**Figure 1:**
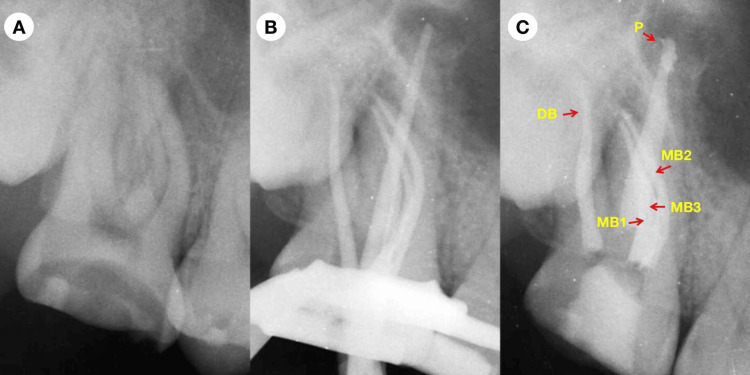
Pre-operative (A), cone-fit (B), and post-obturation (C) radiographs. MB1: mesial buccal first canal; MB2: mesial buccal second canal; MB3: mesial buccal third canal; DB: distal buccal canal; P: palatal canal

Clinical examination revealed normal gingiva around the maxillary right second molar, extensive caries on the occlusal surface, normal tooth mobility, and a negative percussion test. There was a sinus tract on the buccal apical mucosa of the tooth. Periodontal probing results were within normal limits. The maxillary right second molar and healthy control teeth (mandibular right second premolar, mandibular right first molar, and maxillary left second molar) were subjected to a cold test using Endo-Frost cold spray (Roeko, Coltène/Whaledent Inc., Langenau, Germany). The response in the control teeth was characteristic of healthy pulp: a brief, sharp pain that subsided almost immediately after removing the stimulus, indicating normal pulp vitality. In contrast, the maxillary right second molar responded negatively to the cold stimulus. A periapical radiograph showed occlusal decay and possible pulp involvement in the maxillary right second molar, with apparent periapical radiolucency (Figure 1A). The diagnosis for the tooth was necrotic pulp and asymptomatic AP. The treatment options presented to the patient included root canal treatment, no treatment, or extraction of the tooth. The patient opted for root canal treatment. The treatment plan consisted of root canal treatment followed by a full crown restoration for the maxillary right second molar. The patient was informed about and consented to the treatment plan and procedures throughout the pre-operative and entire treatment process. The informed consent form completed by the patient was obtained.

**Figure 2:**
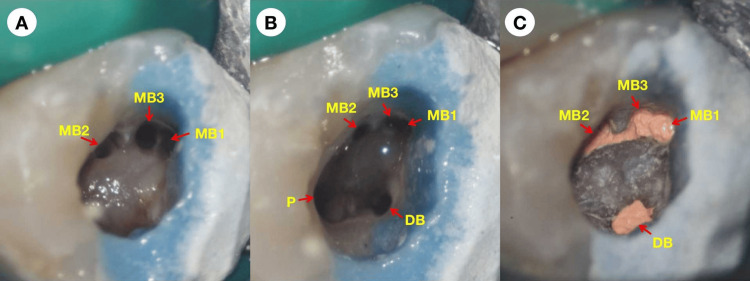
Microscopic photographs reveal that the MB1, MB2, MB3, DB, and P canals have been instrumented (A), irrigated (B), and then filled with gutta-percha and bioceramic sealer (C). MB1: mesial buccal first canal; MB2: mesial buccal second canal; MB3: mesial buccal third canal; DB: distal buccal canal; P: palatal canal

Decay removal and access opening procedures were carried out under a dental operating microscope (M525 F40; Leica, Heerbrugg, Switzerland). The tooth was then isolated with a rubber dam. The pulp chamber walls were refined, and four canals, including the mesial buccal first canal (MB1), mesial buccal second canal (MB2), distal buccal canal (DB), and palatal (P) canal, were located (Figure 2A, 2B). Due to the patient's time constraints, root canal treatment was scheduled for the next appointment. The pulp chamber was dressed with calcium hydroxide paste (Pulpdent™ paste; Pulpdent Corporation, Watertown, USA). A sterile cotton pellet was placed in the pulp chamber, and glass ionomer cement (Fuji IX, GC Corporation, Tokyo, Japan) was used as a temporary filling material to seal the cavity.

The patient presented one week later and was asymptomatic. The tooth was then isolated with a rubber dam. The temporary filling material and the cotton pellet were removed under the dental operating microscope (M525 F40; Leica, Heerbrugg, Switzerland). A copious 1% sodium hypochlorite solution (NaOCl; LangLy Biomedicine CO., Ltd, Wuhan, China) was used to irrigate the pulp chamber to remove calcium hydroxide paste. An ultrasonic tip was used to remove calcification in the pulp chamber floor, and an MB3 canal was located (Figure 2A, 2B). Sizes 8, 10, and 15 K-type files (Dentsply Tulsa Dental, Oklahoma City, USA) were used to negotiate all the root canals. The working length of all canals was determined using an electronic apex locator (Root ZX II; J Morita, Tokyo, Japan). ProTaper Gold (Dentsply Maillefer, Ballaigues, Switzerland) and M3 Pro Gold (United Dental, Shanghai, China) nickel-titanium rotary files driven by an endodontic motor (X-Smart Plus, Dentsply Maillefer) were used to instrument the canals. The speed and torque values of the files were set according to the manufacturer's recommendations. The MB1, MB2, MB3, and D canals were instrumented to F2, while the P canal was instrumented to #50/.04. Copious 1% NaOCl and 17% ethylenediaminetetraacetic acid (EDTA; LangLy Biomedicine CO., Ltd, Wuhan, China) were used to irrigate the canals during instrumentation. Final irrigation procedures were performed using 1% NaOCl and 17% EDTA activated by an ultrasonic file (Satelec Acteon Group, Merignac, France). The canals were dried using absorbent paper points (Gapadent Co Ltd, Tianjin, China). Calcium hydroxide paste (Pulpdent™ paste; Pulpdent Corporation, Watertown, USA) was reintroduced into the canals. A sterile cotton pellet was placed in the pulp chamber, and glass ionomer cement (Fuji IX, GC Corporation, Tokyo, Japan) was used as a temporary filling material to seal the cavity.

The patient presented 10 days later and was asymptomatic. The tooth was then isolated with a rubber dam. The temporary filling material and the cotton pellet were removed under the dental operating microscope. Copious 1% NaOCl activated by an ultrasonic file was used to irrigate the pulp chamber to remove calcium hydroxide paste. Copious 1% NaOCl and 17% EDTA activated by an ultrasonic file were used to irrigate the canals. Once these irrigation steps were completed, the canals were thoroughly cleansed with sterile water and subsequently dried using absorbent paper points (Gapadent Co Ltd, Tianjin, China). A trial fitting of the master gutta-percha cones (Gapadent Co Ltd, Tianjin, China) was then confirmed by a periapical radiograph (Figure 1B). Before placing them, the tips of the gutta-percha cones were dipped in a small amount of AH Plus sealer (Dentsply DeTrey, Konstanz, Germany). The root canal filling was completed using the continuous wave obturation technique (Figure 2C). A periapical radiograph was taken afterward to evaluate the quality of the root canal filling (Figure 1C). The access cavity was temporarily sealed again. The patient was advised to wait two weeks before proceeding with the full crown restoration.

## Discussion

The anatomical complexities of roots and root canal systems in maxillary molars pose significant challenges for endodontic procedures, particularly in locating, cleaning, shaping, disinfecting, and filling the canals [10]. These challenges are compounded by calcification within the pulp chamber, which can obstruct canal access [11-13]. Failure to adequately locate, clean, and shape all root canals can lead to root canal treatment failure, often due to incomplete eradication of bacterial biofilm, resulting in the development or persistence of AP [7,14]. In our case, four canals (MB1, MB2, D, and P) were identified during the initial appointment, although maxillary first molars typically have three canals. Locating an MB2 canal in a maxillary second molar is uncommon in clinical practice. During a subsequent appointment, an MB3 canal was discovered using an ultrasonic tip to remove calcifications under a dental operating microscope, highlighting the importance of ultrasonic techniques and microscopic visualization in effectively locating and treating all canals.

Clinicians need a deep understanding of the anatomical variations of the teeth they are treating to effectively locate all roots and root canals. The MB3 canal, defined as a third main root canal situated between the MB1 and MB2 canals of the mesiobuccal root of maxillary molars, varies widely in prevalence, reported between 0.2% and 4.2% in maxillary second molars [15-25]. This variation is attributed to differences in study designs and demographic factors, as summarized in Table 1.

**Table 1:** Studies reporting the prevalence of MB3 canal in maxillary second molars. MB3: mesial buccal third canal; CBCT: cone-beam computed tomography; micro-CT: micro-computed tomography

Authors (year)	Type of studies	Region	Sample size (n)	MB3 (%)
Lee et al. (2011) [16]	CBCT	Korea	467	0.6
Pérez-Heredia et al. (2017) [17]	CBCT	Spain	112	1.8
Martins et al. (2018) [18]	CBCT	Portugal	589	0.2
Martins et al. (2018) [18]	CBCT	China	189	0.5
Calişkan et al. (1995) [19]	Clearing	Turkey	100	4.2
Alavi et al. (2002) [20]	Clearing	Thailand	65	3.1
Sert & Bayirli (2004) [21]	Clearing	Turkey	200	2.0
Degerness & Bowles (2010) [22]	Cross-sections	USA	63	1.6
Sert et al. (2011) [23]	Clearing	Turkey	252	0.8
Wolf et al. (2017) [24]	Micro-CT	Germany	123	3.3
Ordinola-Zapata et al. (2017) [25]	Micro-CT	Brazil	100	4.0
Ordinola-Zapata et al. (2020) [15]	Micro-CT	Brazil	165	3.6

Illumination and magnification are critical for successfully identifying additional canals. The dental operating microscope has proven effective in locating MB2 canals in maxillary molars. Schwarze et al. conducted a study to determine if using an operating microscope could improve the diagnosis of MB2 canals [26]. Initially, 100 maxillary molars (50 first and 50 second) were examined using ×2 magnifying loupes by Examiner I, followed by examination under an operating microscope with ×8 magnification by a second examiner. The mesiobuccal roots were then separated and analyzed both histologically and by scanning electron microscopy. The histological analysis identified 63 MB2 canals, 39 in first molars and 24 in second molars. Only 26 (41.3%) of these canals were identified using magnifying loupes, whereas 59 (93.7%) were detected with the operating microscope, demonstrating its superior efficacy in identifying these complex anatomical features.

Limited field-of-view CBCT imaging provides three-dimensional insights into the localization of root canal orifices, enabling precise measurements and enhancing procedural accuracy while minimizing tooth damage. Although CBCT is valuable for detailed anatomical explorations, its effectiveness in identifying MB2 canals in maxillary molars has been questioned. Studies by Hiebert et al. and Parker et al. investigated this aspect [27,28]. Hiebert et al. found that MB2 canals were identified in 69% of cases using CBCT alone, increasing to 92% with additional methods like coronal grinding. Parker et al. showed that while initial access located MB2 canals in 70% of cases, the use of CBCT and troughing located MB2 in only 53% of the remaining cases, resulting in an overall detection rate of 86%. Both studies concluded that while CBCT can enhance MB2 detection when canals are not initially found, its standalone use is limited, and combining it with other techniques like a dental operating microscope and troughing can significantly improve outcomes.

Once an MB3 canal is identified in a clinical setting, it requires careful handling due to the thin dentin walls towards the furcation aspect of the mesiobuccal root [15]. A previous micro-computed tomography (micro-CT) study highlighted the risk of root perforation during the preparation of the MB2 canal with large-tapered instruments, due to the limited dentin thickness associated with this canal [29]. Given the similar dentin thickness challenges presented by both the MB2 and MB3 canals, it is advisable to use less tapered instruments for removing dentinal structures during shaping procedures to minimize the risk of perforation [15,30].

## Conclusions

This case report described a rare occurrence of a maxillary second molar with five root canals, including an MB3 canal in the mesiobuccal root. Effective treatment of maxillary second molars, often characterized by complex root variations, necessitates a comprehensive understanding of root canal anatomy. The use of a dental operating microscope is crucial for accurately identifying and treating all root canals.
